# Improving Antioxidative and Antiproliferative Properties Through the Release of Bioactive Compounds From Eucommia ulmoides Oliver Bark by Steam Explosion

**DOI:** 10.3389/fnut.2022.916609

**Published:** 2022-06-30

**Authors:** Feng Kong, Qinghua Zeng, Yue Li, Yishuai Ding, Di Xue, Xingfeng Guo

**Affiliations:** College of Agronomy, Liaocheng University, Liaocheng, China

**Keywords:** steam explosion, network pharmacology, Eucommia ulmoides Oliver, antioxidant, antiproliferation

## Abstract

Eucommia ulmoides Oliver bark is a potential medicinal plant-based feedstock for bioactive products and possesses the effective functions of antioxidant and antitumor. Network pharmacology was employed to reveal the oxidative and free radical damage and cancer-related potential compounds of Eucommia ulmoides Oliver in this study. The result showed that quercetin might be the key compound to resist these two types of diseases. Then, the effect of steam explosion on the release of bioactive compounds and the antioxidative and antiproliferative properties of the extract from Eucommia ulmoides Oliver bark were investigated. Results showed that steam explosion at 0.7 MPa for 30 min significantly enhanced the total phenolic, total flavonoids, and quercetin content of Eucommia ulmoides Oliver bark. Reducing power and 2,2-diphenyl-1-picryl-hydrazyl-hydrate (DPPH) radical scavenging activity of the steam-exploded extracting solution were 1.72 and 2.76 times of native. The antiproliferative activity to CT26 and HepG2 of the extract from steam-exploded Eucommia ulmoides Oliver bark (SEU) was higher than those of native-exploded Eucommia ulmoides Oliver bark (NEU). All these results suggested that steam explosion could be applied to release the bioactive compounds, thus enhanced the antioxidative and antiproliferative activities of medicinal and edible plant-based sources.

## Introduction

Eucommia ulmoides Oliver is a potential feedstock for bioactive products and contains a variety of bioactive compounds, such as phenolics and flavonoids, which possess the functions of antioxidative and antiproliferative activity (1–3). Eucommia ulmoides Oliver bark is a traditional herb, it has been listed in the Chinese Pharmacopeia. The ethanol extracts from Eucommia ulmoides Oliver bark to obtain its bioactive ingredients is widely used as a way of utilization of bioactive parts (4).

Despite the health-promoting factors linked to Eucommia ulmoides Oliver bark, bioactive compounds mainly existed in the cell and cell wall, which limited the release of bioactive ingredients from Eucommia ulmoides Oliver bark into extracting solution. The bioactive compounds are mainly encapsulated in the cells and cell walls of Eucommia ulmoides Oliver bark, and the compositional and structural barriers inevitably restricted the accessibility and dissolution of bioactive ingredients (5, 6). Therefore, for the effective utilization of Eucommia ulmoides Oliver bark, pretreatment before extraction is necessary to enhance the release of bioactive compounds and improve the biological functions.

Steam explosion as an efficient pretreatment method has been widely used to pretreat lignocellulosic materials (7, 8). High-pressure saturated steam was used to pretreat material for a set period of time and then released the pressure instantaneously. The steam explosion broke the cell wall structure, led to an increase in soluble dietary fiber content, and reduced lipase activity and phytic acid of wheat bran (7, 9, 10). Steam explosion significantly increased the ferulic acid content in wheat bran (11) and enhanced the release of bound phenolic compounds of barley bran (12) and soybean seed coat phenolic profiles (13). The steam explosion was a novel hydrothermal processing technology in the food industry with high efficiency and low energy consumption, which was usually employed in high-fiber materials (7, 14, 15).

The key compound that exerted biological function remained largely unexplored as a result of the complexity of the chemical components from Eucommia ulmoides Oliver (4). Network pharmacology is a cost-effective strategy to screen the disease-related bioactive compounds from Chinese herbal medicine, which is based on database information of herb compounds, genes, proteins and diseases, and observed the influence of bioactive compounds on the related diseases (16, 17). The network pharmacology was employed to predict the key compound of Eucommia ulmoides Oliver on oxidation resistance and cancer in this study. In addition, we evaluated the effects of steam explosion on the release of key compounds, the antioxidative (2,2-diphenyl-1-picryl-hydrazyl-hydrate [DPPH] radical scavenging activity and reducing power) and antiproliferative (colon cancer cell line CT26 and liver cancer cell line HepG2) properties of extracts from Eucommia ulmoides Oliver bark.

## Materials and Methods

### Materials and Chemicals

Eucommia ulmoides Oliver bark was purchased from Qiancaolu Chinese Medicine Shop (Bozhou, Anhui, China). Quercetin was purchased from Shanghai Macklin Biochemical Co., Ltd. (Shanghai, China). All other chemicals and solvents were of analytical grade.

### Screening Bioactive Compounds of Eucommia ulmoides Oliver

The active components of Eucommia ulmoides Oliver were retrieved by the Traditional Chinese Medicine Database and Analysis Platform (TCMSP) database (http://www.tcmspw.com/tcmsp.php) (18), and the bioactive compounds were screened based on oral availability (OB) ≥30% and drug-like (DL) ≥0.18 (19). The targets related to oxidative and free radical damage and cancer in the category of protein coding were searched in the GeneCards database (https://www.genecards.org/) with “oxidative and free radical damage” and “cancer” as the keywords (20). The Cytoscape 3.8.2 software (Bethesda, MD, USA) was employed to establish a compound-target network (21).

### Steam Explosion Process

The steam explosion was performed in a self-designed batch vessel, which mainly consisted of a reactor chamber (WY19, Big Soldier Food Machinery, Henan, China) and a steam generator (WY19, Big Soldier Food Machinery, Henan, China). Eucommia ulmoides Oliver bark was infiltrated by distilled water with a ratio of water to materials 1:2 (w/w) on dry weight of Eucommia ulmoides Oliver bark. The Eucommia ulmoides Oliver bark was loaded into a reactor chamber from the feed valve, then closed the feed valve, and charged high-pressure saturated steam to the reactor chamber. The reactor pressure was raised and then maintained at 0.7 MPa for 10–40 min, respectively. Afterward, the reaction system was then terminated with Eucommia ulmoides Oliver bark suddenly exploded by opening the feed valve. The samples were collected and dried in the oven at 60°C for 12 h.

### Determination of Bioactive Compounds

Native- and steam-exploded Eucommia ulmoides Oliver bark (NEU and SEU) powder (2 g) was dispersed, respectively in petroleum ether (20 ml) and ethanol (20 ml) and centrifugated at 3,000 rpm for 10 min, repeated the above steps three times. The extracting solution was obtained by collecting the supernatant and concentrating at 10 ml. Total phenolics content (TPC) of Eucommia ulmoides Oliver bark extracting solution was measured by the Folin-Ciocalteu colorimetric method (22). Total flavonoids content (TFC) of Eucommia ulmoides Oliver bark extracting solution was analyzed by the method (23). The extracting solution (0.2 ml) was added to 10 ml of ethanol, the quercetin content of Eucommia ulmoides Oliver bark extracting solution was determined by spectrophotometric method at 370 nm (24). Three replicate tests were carried out and the average values were reported.

### Antioxidant Activity of Eucommia ulmoides Oliver Bark Extracting Solution

The 2,2-diphenylpicrylhydrazyl (DPPH) radical scavenging activity of Eucommia ulmoides Oliver bark extracting solution was measured according to the method (7). The extracting solution (50 μl) was added to 0.1 mmol/L of DPPH methanol solution (2 ml), the mixture reacted in the dark, and was measured at 517 nm. The reducing power of extracting solution was determined according to Tu et al. (25) with modifications. The extracting solution (1 ml) was mixed with 1% of potassium ferricyanide (2.5 ml) and 0.2 mol/L of phosphate buffer (2.5 ml, pH 6.6), and the mixture was incubated at 50°C for 20 min. After adding 10% of trichloroacetic acid (2.5 ml), the mixture (2.5 ml) was added to 2.5 ml of distilled water and 2.5 ml of 0.1% ferric chloride, homogeneously mixing and standing for 10 min, and measured at 700 nm. The increased absorbance of the mixture solution indicated reducing power. Three replicate tests were carried out and the average values were reported.

### Antiproliferative Activity of Eucommia ulmoides Oliver Bark Extracts

The Eucommia ulmoides Oliver bark extracting solution was freeze-dried to obtain the extract. The antiproliferation of Eucommia ulmoides extracts was tested on CT26 and HepG2 cell lines for toxicity. Stock solutions of Eucommia ulmoides Oliver bark extracts were diluted to 1, 2, 4, 6, and 8 mg/ml. Each dilution of 100 μl was transferred into the 96-well-plate when the confluency reached 60%. A blank control was also performed at the same time. The plate was placed in the MCO-15AC carbon dioxide incubator under the condition of 37°C, 5% CO_2_. 3-(4,5-dimethylthiazol-2-yl)-2,5-diphenyl tetrazolium bromide (MTT) assay was used to detect the effects of eucommia ulmoides extract on CT26 and HepG2 cell viability. The 96-well-plate was read at 490 nm after being shaken with the iMark Microplate Reader (BioTek, USA). Three replicate tests were carried out and the average values were reported.

### High-Performance Liquid Chromatography Mass Spectrometry(HPLC-MS) Analysis of Eucommia ulmoides Oliver Bark Extracting Solution

High-performance liquid chromatography mass spectrometry analysis was performed on Waters G2-XS Q-TOF. Bioactive compound separation was accomplished on a Waters ACQUITY UPLC I-Class, this instrument was equipped with a Waters C18 column (BEH C18 2.1 × 50 mm, 1.7 μm) under 25°C. Acetonitrile/water (90:10) was designated as the mobile phase. The HPLC flow rate was set at 0.4 ml/min, the injection volume was 1 μl.

## Results and Discussion

### Prediction of Potential Antioxidative and Antiproliferative Compounds of Eucommia ulmoides Oliver

**Figure 1:**
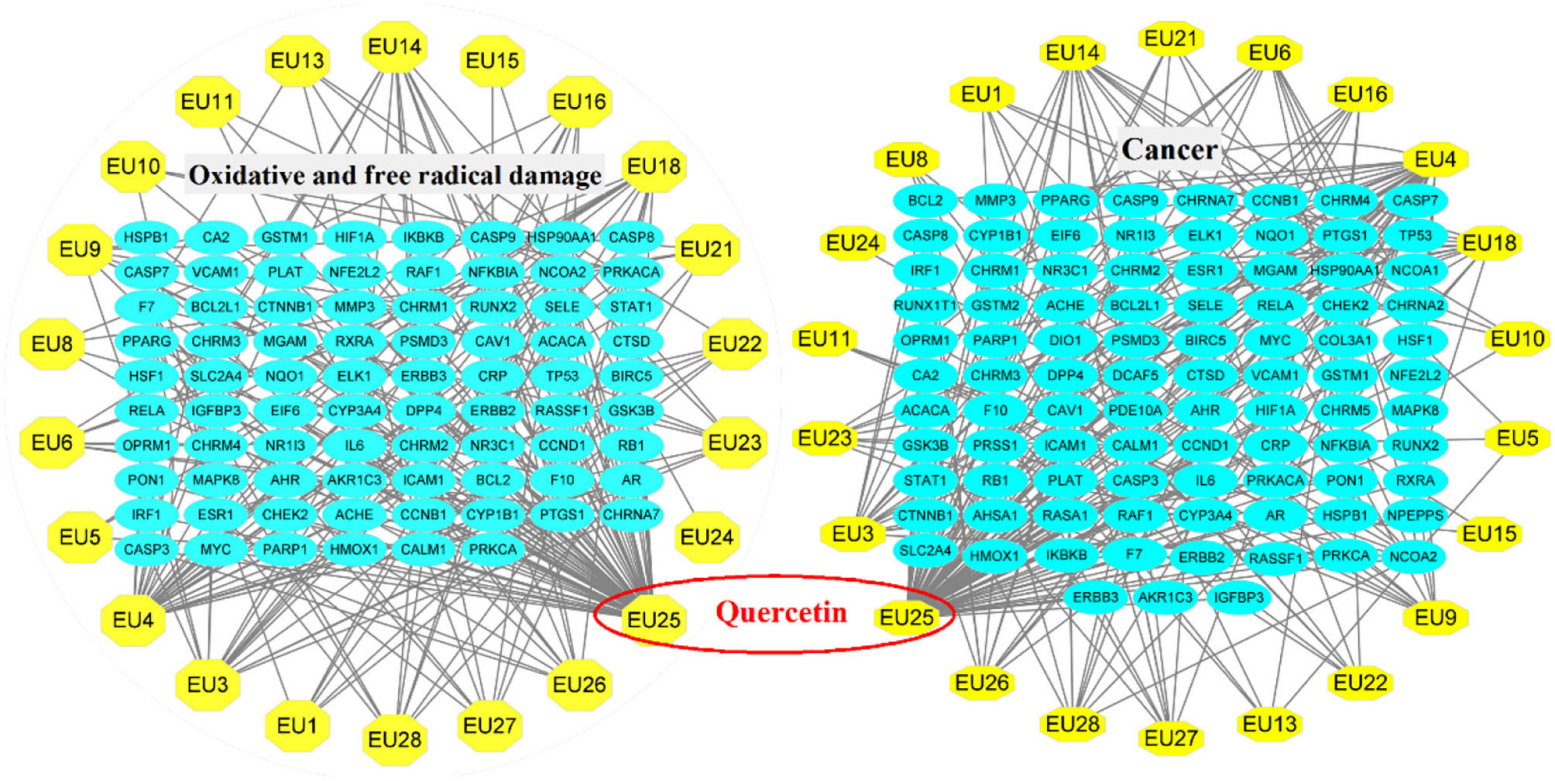
The network of compound-target interactions.

There were 28 compounds of Eucommia ulmoides Oliver through TCMSP screened under the conditions of OB ≥30% and DL ≥0.18 (Table 1). The chemical compounds of Eucommia ulmoides Oliver included flavonoids, lignans, terpenoids, alkaloids, iridoids, and other compounds. Flavonoids is an important secondary metabolite, such as quercetin and kaempferol and possesses the functions of antioxidant and anti-inflammatory. Lignans are the main chemical components in Eucommia ulmoides Oliver, which mainly existed in bark (26). The pharmacological action of Eucommia ulmoides Oliver has gotten the attention due to the chemical compounds, which have been used in antioxidative and antitumor (4, 27). According to TCMSP and UniPort databases, there were 258 targets for the active components of Eucommia ulmoides Oliver. In the GeneCards database, 3,012 related targets of oxidative and free radical damage, 26,082 cancer-related targets were retrieved, which were taken as candidate target genes for the disease. The network diagram of compound-targets was made by using Cytoscape 3.8.1. After the intersection of 28 compound-related targets and oxidative and 78 free radical damage-related targets, 91 cancer-related targets were obtained, the 22 compounds are shown in Figure 1. The ellipse node represented disease targets and the octagon node represented the compounds of Eucommia ulmoides Oliver.

**Table 1:** Pharmacokinetic properties of candidate compounds of Eucommia ulmoides Oliver.

**Number**	**Molecule name**	**MW**	**OB (%)**	**DL**
EU1	Medioresil	388.45	57.20	0.62
EU2	Mairin	456.78	55.38	0.78
EU3	beta-sitosterol	414.79	36.91	0.75
EU4	Kaempferol	286.25	41.88	0.24
EU5	Olivil	376.44	62.23	0.41
EU6	Erythraline	297.38	49.18	0.55
EU7	acanthoside B	580.64	43.35	0.77
EU8	8-hydroxypinoresinol	374.42	92.43	0.55
EU9	3-beta-Hydroxymethyllenetanshiquinone	294.32	32.16	0.41
EU10	ent-Epicatechin	290.29	48.96	0.24
EU11	Yangambin	446.54	57.53	0.81
EU12	Eucommin A	550.61	30.51	0.85
EU13	(+)-medioresinol	388.45	87.19	0.62
EU14	(-)-Tabernemontanine	354.49	58.67	0.61
EU15	Cyclopamine	411.69	55.42	0.82
EU16	Dehydrodiconiferyl alcohol 4,gamma'-di-O-beta-D-glucopyanoside_qt	358.42	51.44	0.40
EU17	Dehydrodieugenol	326.42	30.10	0.24
EU18	Cinchonan-9-al, 6'-methoxy-, (9R)-	324.46	68.22	0.40
EU19	GBGB	550.57	45.58	0.83
EU20	Helenalin	262.33	77.01	0.19
EU21	(+)-Eudesmin	386.48	33.29	0.62
EU22	4-[(2S,3R)-5-[(E)-3-hydroxyprop-1-enyl]-7-methoxy-3-methylol-2,3-dihydrobenzofuran-2-yl]-2-methoxy-phenol	358.42	50.76	0.39
EU23	hirsutin_qt	345.35	49.81	0.37
EU24	liriodendrin_qt	450.48	53.14	0.80
EU25	quercetin	302.25	46.43	0.28
EU26	beta-carotene	536.96	37.18	0.58
EU27	(E)-3-[4-[(1R,2R)-2-hydroxy-2-(4-hydroxy-3-methoxy-phenyl)-1-methylol-ethoxy]-3-methoxy-phenyl]acrolein	374.42	56.32	0.36
EU28	Syringetin	346.31	36.82	0.37

*MW, molecular weight; OB, oral availability; DL, drug-like*.

The topological analysis and network diagram of compounds-targets are shown in Figure 1. The degree of nodes (the number of wires between a node and other nodes) as a significant index was used to judge the importance of nodes. The larger the degree of quercetin, the more vital it was in the network, and the more biological functions they participated in (20). The oral availability of quercetin is 46.43%, and the DL is 0.28. Quercetin was reported to be found in almost all plants and was one of the most abundant flavonoids in the human diet (28). Quercetin contributed to the antioxidant activity of fresh lychee pulp (29), caused significant apoptosis, and reduced tumor cell proliferation (30).

### The Release of Bioactive Compounds From Eucommia ulmoides Oliver Bark

**Figure 2:**
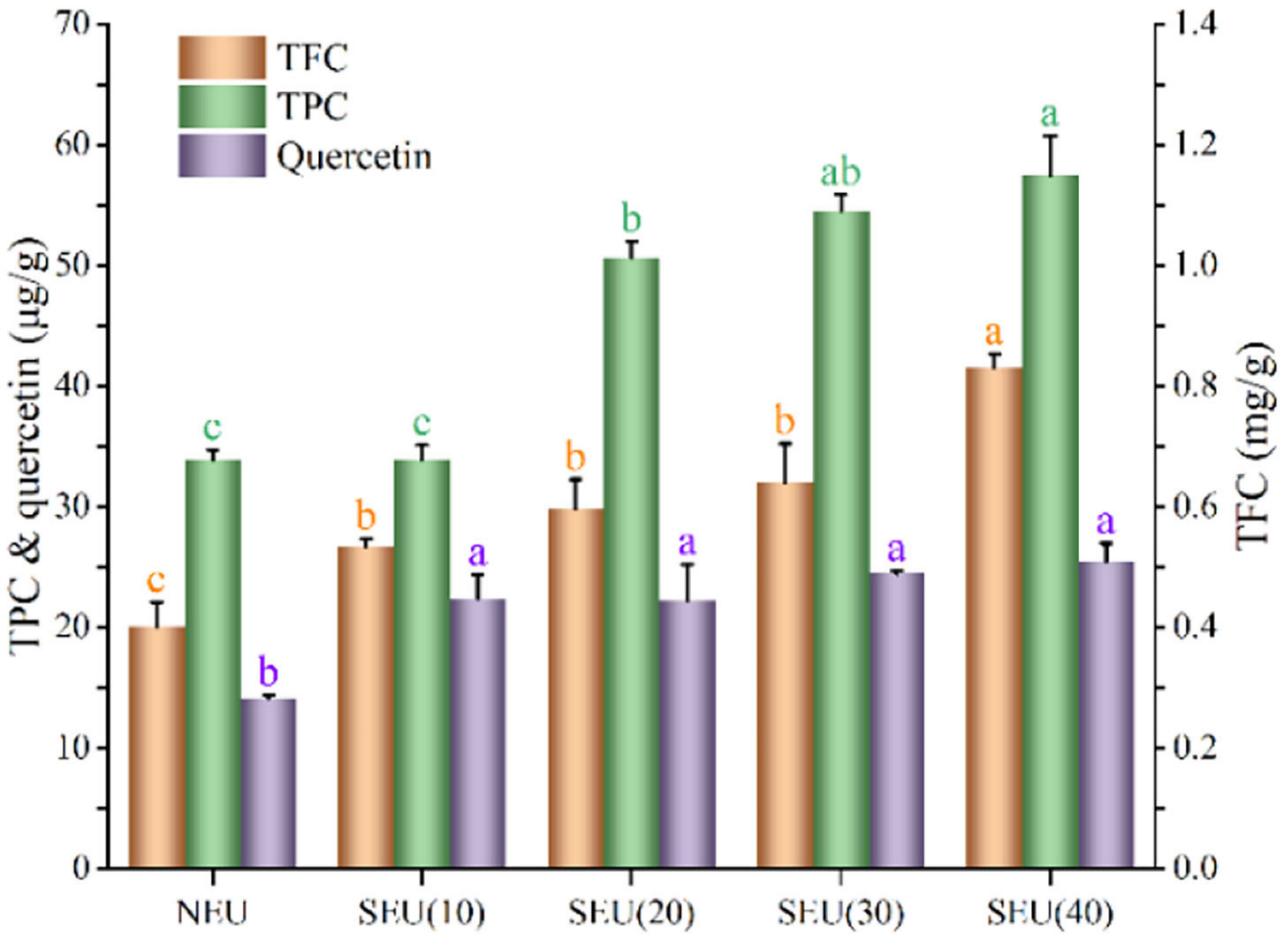
Effect of steam explosion on bioactive compounds of Eucommia ulmoides Oliver bark. TFC, total flavonoids content; TPC, total phenolics content; NEU, native Eucommia ulmoides Oliver bark; SEU, steam-exploded Eucommia ulmoides Oliver bark, SEU(10) to SEU(40) indicated steam explosion retention time was 10–40 min, respectively. (Means that do not share a letter are significantly different, *p* < 0.05). Different letters in data bars represent significant differences at *p* < 0.05 as determined by Duncan's multiple range test.

Phenolics could increase the reactive antioxidant potential and subsequently decrease the risk of free radical-related diseases, they also possessed the functions, such as anti-inflammatory and antitumor activities (4, 31). The steam explosion at 0.7 MPa for 20, 30, and 40 min significantly (*p* < 0.05) enhanced TPC, which was 1.49, 1.61, and 1.69 times of those native bark, respectively. Steam explosion significantly (*p* < 0.05) increased the quercetin content in the Eucommia ulmoides Oliver bark at 0.7 MPa for 10–40 min. The release of quercetin in the extract from Eucommia ulmoides Oliver bark treated by the steam explosion is shown in Figure 2. After being treated by steam explosion, quercetin content in extracts was significantly increased (*p* < 0.05). In the steam explosion process, the hydrolysis reactions were carried out under mild acidic conditions, which come from a decrease of water pK_w_ at high temperatures and the release of organic acids from steam-penetrated feedstock (32). The effects were partly responsible for steam explosion that facilitated the major destruction of the cell wall of Eucommia ulmoides Oliver bark. Bioactive compounds were encapsulated from intact cells and steam explosion could have a significant effect of the high shear and high temperatures on cell integrity and bioaccessibility. The cell wall structure of Eucommia ulmoides Oliver bark was seriously damaged by steam explosion, which resulted in reducing the diffusion resistance and improving the efficiency of mass transfer. Therefore, the ethanol solvent might be more easily permeated to the bark materials and increased the amount of flavonoids and phenolics in the ethanol solution (6). The mechanical action of the steam explosion caused the exposure of internal substances in Eucommia ulmoides Oliver bark, improved the accessibility of bioactive compounds in cell wall (11, 12), which may lead to the bioactivity improvement of the Eucommia ulmoides Oliver bark extract.

### Antioxidant Capacity of Eucommia ulmoides Oliver Bark Extracting Solution

**Figure 3:**
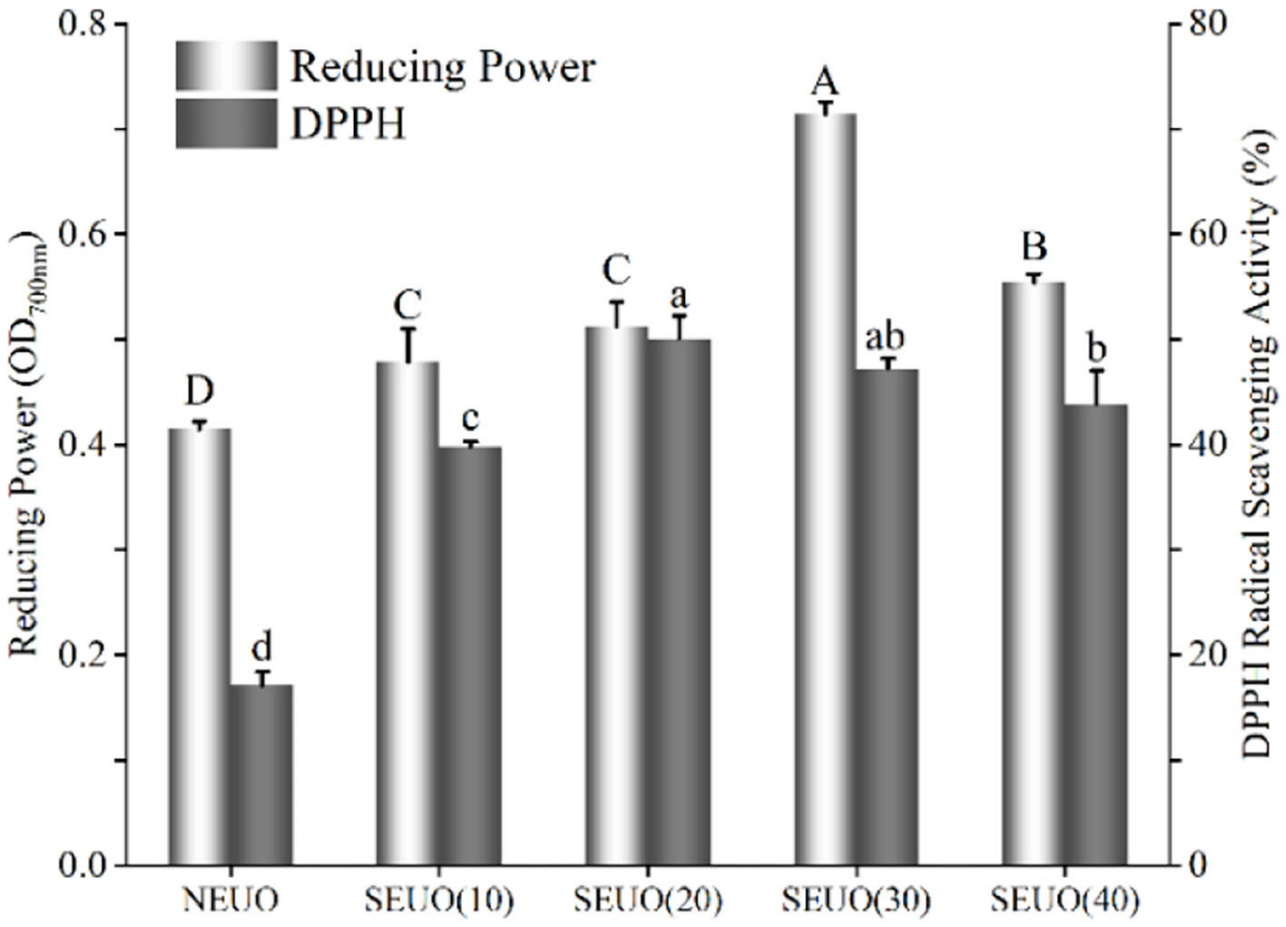
Effect of steam explosion on antioxidant capacity of Eucommia ulmoides Oliver bark extracting solution. DPPH, DPPH radical scavenging activity; NEU, native Eucommia ulmoides Oliver bark; SEU, steam-exploded Eucommia ulmoides Oliver bark, SEU(10) to SEU(40) indicated steam explosion retention time was 10–40 min, respectively. (Means that do not share a letter are significantly different, *p* < 0.05). Different letters in data bars represent significant differences at *p* < 0.05 as determined by Duncan's multiple range test.

2,2-diphenyl-1-picryl-hydrazyl-hydrate radical scavenging activity and reducing power assays were employed to compare the antioxidant activity of the extracting solutions from NEU and SEU (Figure 3). It demonstrated that the DPPH radical scavenging activity of the steam-exploded samples was stronger (*p* < 0.05) than those of native. DPPH radical scavenging activity tended to be firstly increased and then decreased along with the increase of retention time. When the steam explosion retention time increased from 10 to 20 min, DPPH radical scavenging activity was increased to reach the maximum value of 50.09%, which was 1.93-fold higher than that of native. There was no difference (*p* > 0.05) between the conditions at 0.7 MPa for 20 and 30 min, then it began to decrease with the further increase of retention time. The reducing power values of the steam-exploded extracting solutions at 10–40 min were 0.48, 0.51, 0.71, and 0.55, respectively, the reducing power of the native extracting solution was only 0.41. The absorbance of the steam-exploded extracts was higher than that of native, which suggested that steam-exploded extracting solutions had excellent antioxidant activity. At 0.7 MPa for 30 min, the reducing powder reached the maximum to 0.71, which was 1.72-fold of native. The result coincided with the present results, which was found that steam explosion enhanced the antioxidant activity of the extract from wheat bran (7, 9, 11). The steam explosion had the effect of breaking the cell wall, which aided the release of phenolic profiles and thus enhanced the antioxidant activities (7, 13, 33).

### Antiproliferative Activity of Eucommia ulmoides Oliver Bark Extracts

**Figure 4:**
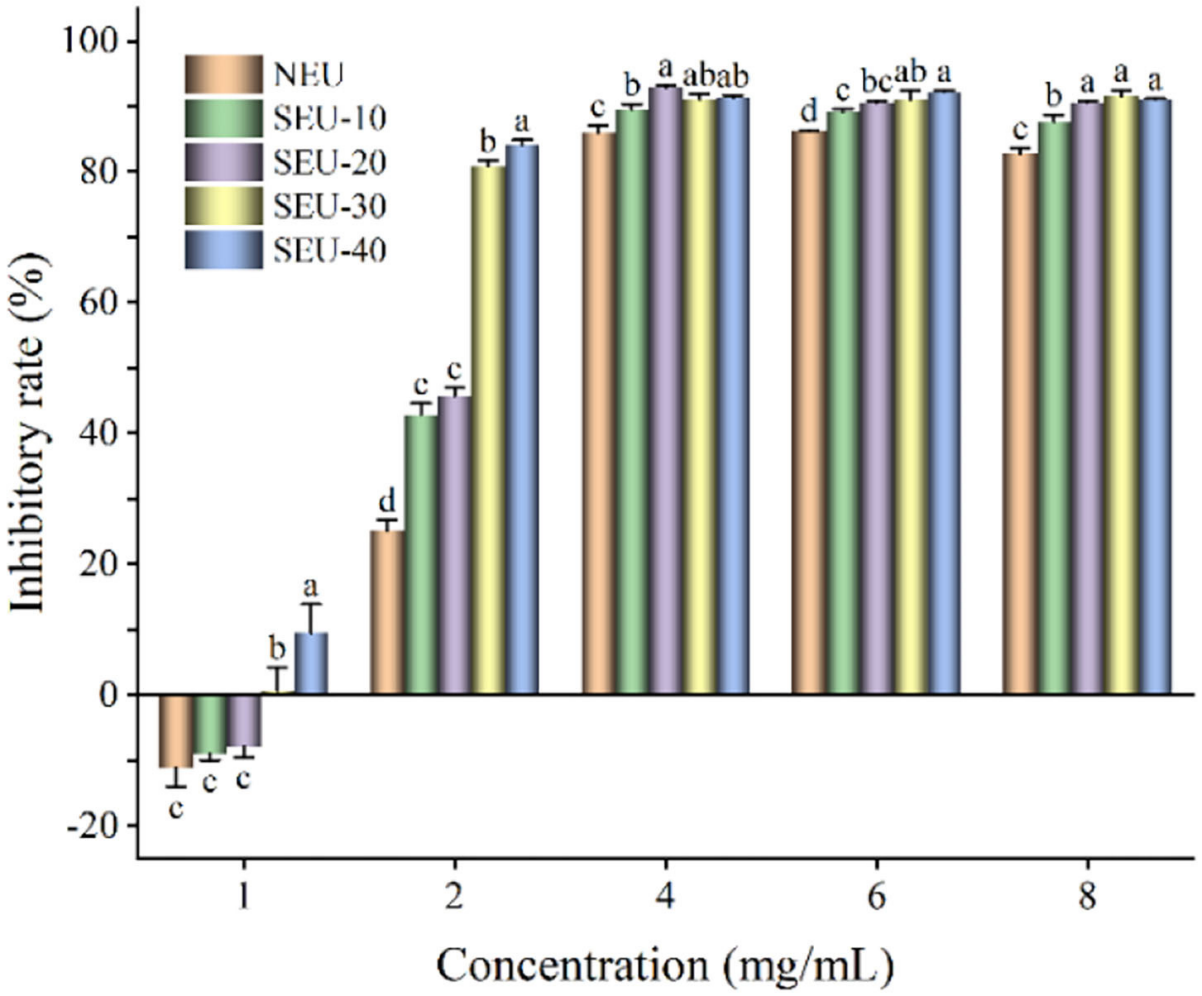
Inhibitory rate of Eucommia ulmoides Oliver bark extract on CT26 cells. NEU, native Eucommia ulmoides Oliver bark; SEU, steam-exploded Eucommia ulmoides Oliver bark, SEU(10) to SEU(40) indicated steam explosion retention time was 10–40 min, respectively. (Means that do not share a letter are significantly different, *p* < 0.05). Different letters in the same concentration indicate significant differences at *p* < 0.05 as determined by Duncan's multiple range test.

The colon cancer cell line CT26 and liver cancer cell line HepG2 were employed to evaluate the inhibitory effect of Eucommia ulmoides Oliver bark extracts on the cancer cell proliferation. The CT26 cells were incubated with Eucommia ulmoides Oliver bark extracts in an ascending concentration for 24 h. It was found that the Eucommia ulmoides Oliver bark extracts exhibited a better anticolon cancer effect and thus decreased viability of CT26 cells in a concentration-dependent manner (Figure 4). The CT26 cell viability of steam-exploded extracts was significantly lower than that of native extract. However, when extract concentration was 1 mg/ml, the inhibitory rates of native and steam-exploded extracts at 0.7 MPa for 10–20 min on CT26 cells were −11.14, −9.02, and −7.91%, respectively. The CT26 proliferation was improved in these conditions, and the antiproliferation was improved when the extract was more than 1 mg/ml. It showed that the extract had the characteristics of low concentration promotion and high concentration inhibition on CT26 proliferation. The result coincided with phenolics that had a significant inhibitory effect on the butanol fermentation, when soluble lignin compounds concentration in steam-exploded corn stover exceeded to 1.77 g/L (34). In the comparison of all extracts, the 4 mg/ml of extract from Eucommia ulmoides Oliver bark treated by steam explosion at 0.7 MPa for 30 min, with lower energy consumption and greater inhibition effect on cell viability of CT26 cells.

**Figure 5:**
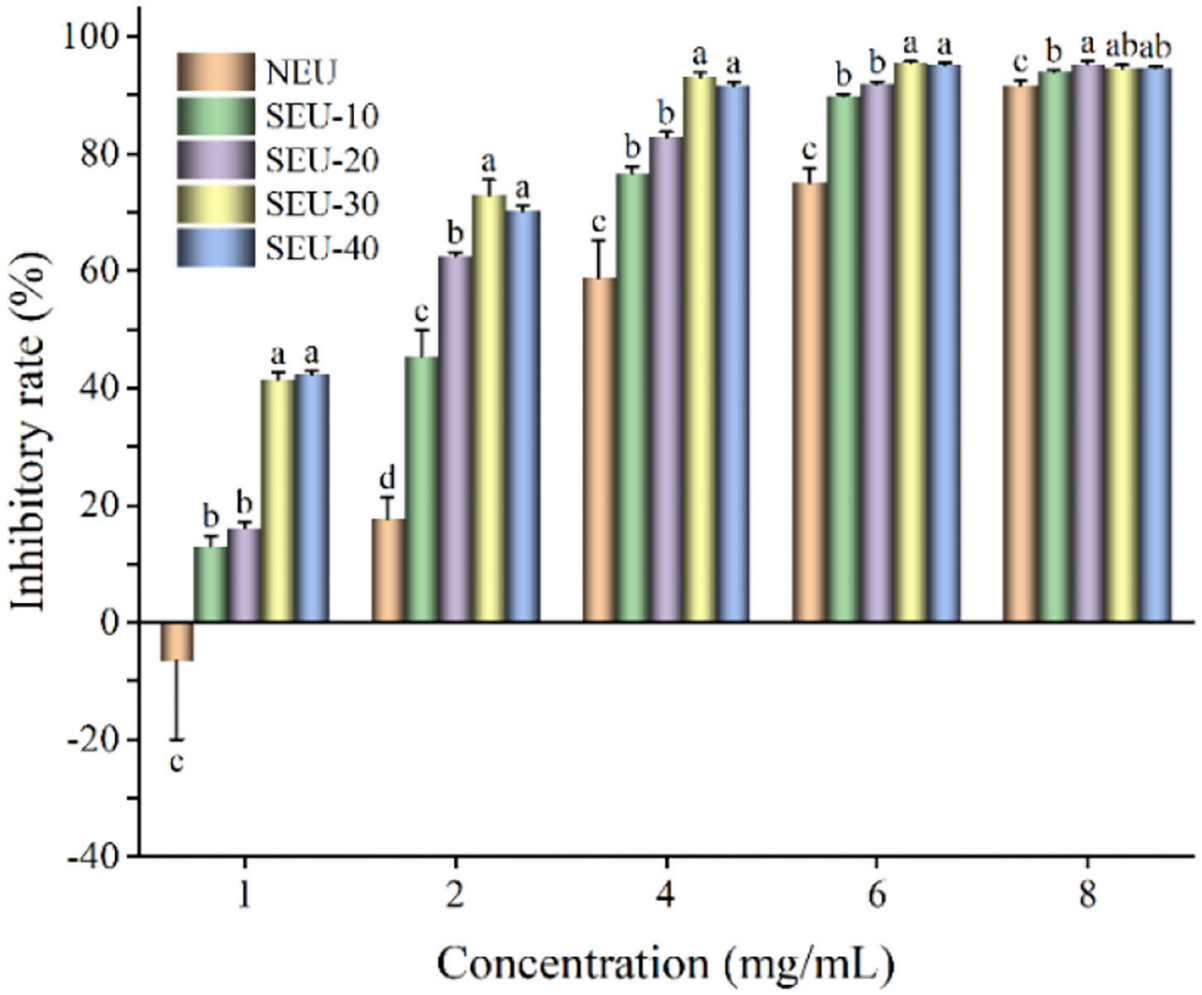
Inhibitory rate of Eucommia ulmoides Oliver bark extract on HepG2 cells. NEU, native Eucommia ulmoides Oliver bark; SEU, steam-exploded Eucommia ulmoides Oliver bark, SEU(10) to SEU(40) indicated steam explosion retention time was 10–40 min, respectively. (Means that do not share a letter are significantly different, *p* < 0.05). Different letters in the same concentration indicate significant differences at *p* < 0.05 as determined by Duncan's multiple range test.

The effects of NEU and SEU extracts on antiproliferation of HepG2 cell lines were evaluated under different concentrations (Figure 5). With the increase in the extract concentration, the inhibitory rate of HepG2 cells was increased significantly. Compared with native extract, high antiproliferation of HepG2 cell was obtained in the steam-exploded extract from 4 to 8 mg/ml. In the comparison of all extracts, the 4 mg/ml of extract from Eucommia ulmoides Oliver bark treated by the steam explosion at 0.7 MPa for 30 min had a greater inhibition effect on the cell viability of CT26 cells. Reports showed that the extract of wheat bran after the steam explosion could greatly improve the antiproliferation to HepG2 (11). The extract from steam-exploded bark of all the concentrations indicated the high antiproliferative activities in a dose-dependent manner. The strong toxicity of bioactive compounds extracted from Eucommia ulmoides Oliver bark toward the HepG2 cell might be due to their aromatic properties that easily penetrated cell membranes, resulted in a decrease in cell growth (35).

### Correlation of Bioactive Compounds and Functions of Eucommia ulmoides Oliver Bark

The correlation coefficients between bioactive compounds (TPC, TFC, and quercetin content) and functions (reducing power, DPPH radical scavenging activity, CT26, and HepG2 inhibitory activity) of extracts from Eucommia ulmoides Oliver bark are shown in Table 2. A positive correlation (*p* < 0.05) was found between the TFC and the CT26 inhibitory activity of 1 mg/ml (r = 0.940), 2 mg/ml (r = 0.901), and 6 mg/ml (r = 0.936) of extract. Moreover, the TPC was positively (*p* < 0.05) related to the CT26 inhibitory activity of 6 mg/ml (r = 0.891) and the HepG2 inhibitory activity of 2 mg/ml of extract (r = 0.895). Quercetin content exhibited significant (*p* < 0.05) correlations with the DPPH radical scavenging activity (r = 0.908) and the CT26 inhibitory activity of 6 mg/ml (r = 0.963) and 8 mg/ml (r = 0.949) of extract. Quercetin had the strongest correlation (*p* < 0.05) with the HepG2 inhibitory activity.

**Table 2:** Correlation coefficients of phytochemicals and bioactivities of extracts.

**Correlation**	**RP**	**DPPH**	**CT26-1**	**CT26-2**	**CT26-4**	**CT26-6**	**CT26-8**	**HepG2-1**	**HepG2-2**	**HepG2-4**	**HepG2-6**	**HepG2-8**
**coefficients (r)**												
TPC	0.558	0.692	0.940*	0.901*	0.700	0.936*	0.831	0.904*	0.855	0.875	0.835	0.731
TFC	0.738	0.723	0.826	0.872	0.771	0.891*	0.871	0.874	0.895*	0.877	0.782	0.749
Quercetin	0.709	0.909*	0.731	0.857	0.840	0.963**	0.949*	0.909*	0.948*	0.964**	0.991**	0.891*

TFC, total flavonoids content; TPC, total phenolics content; RP, reducing power; DPPH, DPPH radical scavenging activity. Statistics: ^*^p < 0.05, ^**^p < 0.01.

### Qualitative Analysis of SEU Extracting Solution

**Figure 6:**
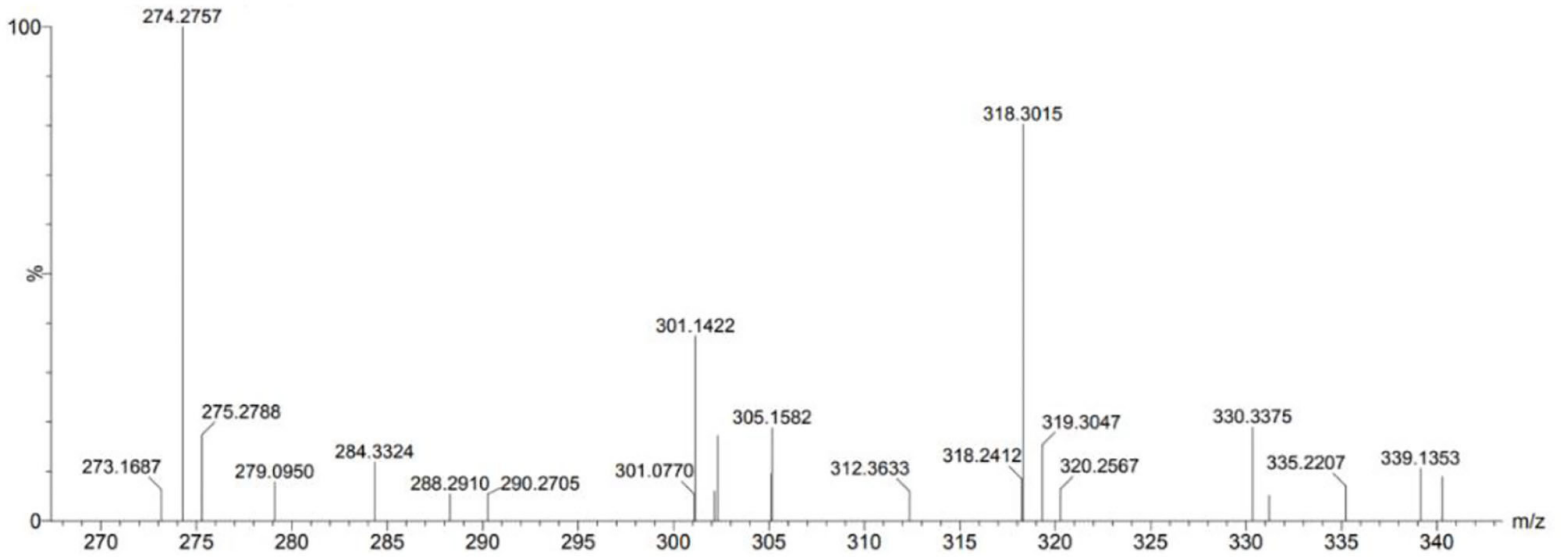
Chromatogram from steam-exploded Eucommia ulmoides Oliver bark extracting solution.

The HPLC-MS method was used to evaluate the bioactive compounds present in SEU extracting solution at 0.7 MPa for 30 min. As shown in Figure 6, the compounds detected in this work are interpreted by the observed MS spectra when compared with those found in the literature (36, 37). The Eucommia ulmoides Oliver bark extracting solution might include phenyl laurate, luteolin, catechin, quercetin, periplobiose, and coumaroylquinic acid. These compounds had the potential to have bioactivity (4).

## Conclusion

The steam explosion could remarkably improve the total phenolics and flavonoids content of Eucommia ulmoides Oliver bark. The quercetin content in the Eucommia ulmoides Oliver bark was increased after steam explosion treatment. The antioxidant activity of the steam-exploded bark extracting solution was higher than that of the native. The antiproliferative activity of the steam-exploded bark extract was significantly improved. Results indicated that the Eucommia ulmoides Oliver bark treated by the steam explosion had the potential nutraceuticals preparations. The steam explosion can be used to improve the bioactive compositions and bioactivity of medicinal and edible plant-based sources.

## Data Availability Statement

The original contributions presented in the study are included in the article/supplementary material, further inquiries can be directed to the corresponding authors.

## Author Contributions

FK and XG: conceptualization, resources, and supervision. FK, YL, and QZ: methodology and validation. FK: software, formal analysis, data curation, writing—original draft preparation, writing—review and editing, project administration, and funding acquisition. QZ, FK, YL, YD, and DX: investigation. QZ and FK: visualization. All authors have read and agreed to the published version of the manuscript.

## Funding

This study was financially supported by the Open Project of Liaocheng University Animal Husbandry Discipline (No. 319312101-08), the Doctoral Research Startup Foundation of Liaocheng University (No. 318052122), and the Project of Shandong Province Higher Educational Science and Technology Program for Youth (No. 2019KJF028).

## Conflict of Interest

The authors declare that the research was conducted in the absence of any commercial or financial relationships that could be construed as a potential conflict of interest.

## Publisher's Note

All claims expressed in this article are solely those of the authors and do not necessarily represent those of their affiliated organizations, or those of the publisher, the editors and the reviewers. Any product that may be evaluated in this article, or claim that may be made by its manufacturer, is not guaranteed or endorsed by the publisher.
